# Atlanta Academy of Medicine

**Published:** 1875-04

**Authors:** Robert Battey

**Affiliations:** Reporter


### Atlanta Academy of Medicine.

ROBERT BATTEY, M.D., REPORTER.

ATLANTA, GA., 23d February, 1875.

Dr. V. H. Taliaferro, Vice-President, presiding.

Dr. Conally had seen recently a case which he considered varioloid. He desired to know if any other cases had occurred. His patient had come recently from Athens, where varioloid now prevails.

Dr. Battey had seen a suspicious case, which afterwards turned out to be rubeola.

Drs. W. F. Westmoreland and Boring had each seen suspicious cases, which proved to be varicella.

Dr. Calhoun had observed that varicella, in three or four epidemics, both at home and abroad, had prevailed either just before or just after variola. Is there any connection or relationship between the two diseases?

Dr. J. G. Westmoreland had not himself observed the coincidence.

Dr. Taliaferro had observed such a coincident occurrence during the epidemic of small-pox at Columbus.

Drs. Boring, Holmes and Conally had observed the same.

Dr. Armstrong had not observed the connection.

Dr. Battey reported the death of his case of ovariotomy on the 13th day, of broncho-pneumonia, there being a prevalent epidemic of pneumonia and influenza in the city. At the last meeting of the Academy she was doing well, as reported. Upon the following day she appeared to have taken cold, and had a troublesome irritative cough. On the 10th day there was observed for a few hours the brick-dust sputum of pneumonia, quickly followed by a rather profuse bronchorrhœa. On the 11th day the expectoration assumed a yellowish cast, was free and copious. On the morning of the 12th day she still held up well, but about noon began to give way. At 6 P.M. the right lung, which was alone implicated, was greatly choked up with mucus, which was expectorated imperfectly and with difficulty. He remained, freely stimulating her, until 11 P.M., when the respiration and pulse were better, and feeling assured that she would survive the night, he left her in charge of Dr. J. W. Clements, of Chattooga county, who had kindly volunteered his services. She gradually sank, and expired in the latter part of the night, the immediate cause of death being apnœa. The autopsy was made by Dr. Baird, who will report his observations to the Academy, Drs. Westmoreland, Holmes, Taliaferro, Ray and Kendrick being present. As the general symptoms and auscultation left no doubt of the accuracy of the diagnosis of broncho-pneumonia, involving the whole of the right lung and not extending to the left, and in consideration of the fact that the body was to be promptly returned to the family by rail, no inspection of the lungs was allowed.

Dr. Baird reported the autopsy made ten hours after death. The edges of the incision had united firmly, and were closely adherent to the omental stump, which had been brought out at the upper angle, and also to the pedicle lying in the lower angle of the incision. A flap of the abdominal wall was formed and turned down. The inner surface was somewhat discolored for several inches in extent on each side of the incision, presenting a bruised appearance. This is accounted for by the infiltration of the subserous areolar tissue by a small quantity of blood during the operation for removal of the tumor, and the hyperæmia of the parietal peritoneum. An adhesion had formed between the omentum and the abdominal wall around the point at which the omental stump emerged. While separating this limited adhesion a circumscribed abscess, containing about two drachms of laudable pus, was encountered; also another near the same spot in process of formation, much smaller, and as yet containing no pus. The peritoneum was injected, markedly so in the pelvic cavity, and that portion forming the right broad ligament of the uterus was intensely congested. Recent adhesions existed between several coils of the small intestine and the peritoneum covering the bladder. A firmer, though evidently recent, adhesion was observed between a very small portion of the sigmoid flexure and some part of the peritoneum on the left side of the cavity. Old adhesions were found between several coils of intestine and the inferior portion of the left broad ligament. There was no fluid in the cavity, and, with the exception of the recent adhesions and the little abscesses noted above, no indication of peritonitis was found; for, in serous membranes, it matters not how intense the hyperæmia may be, inflammation cannot be said to exist without evidence afforded by inflammatory products. The post-mortem staining of the tissues by hæmatin, the coloring matter of the red globules, must also be borne in mind. The right ovary appeared to be healthy.

Dr. W. F. Westmoreland reported a case where amputation of the thigh became necessary in consequence of hemorrhages from the popliteal artery. He presented the case more to illustrate the effects of the muriated tinct. of iron in arresting hemorrhage from the larger arteries than from any peculiarity or interesting feature in the amputation itself. The case was that of a lady twenty-eight years old, who had suffered with a disease of the tibia for twenty years. Numerous pieces of bone had been removed at different periods without any permanent benefit. The disease continued, and in the past year became so extensive and painful it was thought best to amputate the leg, and the amputation was made in Louisville, in July, 1874, by some surgeon of that city. In October, three and a half months after the operation, I was called to see the case, and found an extensive ulceration of the anterior flap, presenting a phagadenic appearance, and rapidly extending itself. The bones of the leg were at this time extensively involved, presenting the appearance of caries rather than necrosis. From the appearance of the stump he was impressed with the idea that he had something similar to the cases so frequently met with the last two years of the late war, and upon that idea it was treated with nitric acid thoroughly applied, followed by compression after the eschar was thrown off. The patient was not materially benefited by this and various other plans of treatment that he adopted during the first two or three months. Sometimes the ulcerative process was arrested for a few days to return with more violence. The idea of hereditary syphilis as the origin of the trouble was acted on by the use of large doses of the iodide of potassium, but without results. Many of the features of malignant disease were wanting. The disease continued to extend, until the flaps were all destroyed and the bones and entire soft parts to near the knee joint. About the 20th of January ult. the popliteal artery gave way, and she had a hemorrhage to syncope. He saw her and applied compression and iron, without cleaning the stump, as he did not have adequate assistance. Two days later she had another hemorrhage, and being quite sick himself, he had Dr. Battey to see the case, and he arrested the hemrrhage for the time by the application of the tourniquet. Twelve or fourteen hours later he saw her with Dr. B., and, upon examination of her condition, it was decided that in her then state she would not survive an amputation, and even an attempt to ligate the artery would be extremely hazardous. The artery was compressed, the tourniquet removed, the stump thoroughly cleansed, and the disorganized tissue removed with a pair of scissors, until the artery was exposed. The end of the artery and surrounding tissues were saturated with the mur. tinct. iron for ten or fifteen minutes, and then covered with lint saturated likewise with the same styptic, and secured in that position by adhesive plaster and a roller. This mode of dressing was practised every second day for two weeks, without the loss of one drop of blood, and, but for the fact that the ulceration was still going on, Dr. W. was of the impression that nothing further would have been necessary to arrest the flow from the artery. At the end of two weeks more the patient had sufficiently recuperated to bear the amputation above the knee. Esmarch's elastic tourniquet was applied, and the thigh amputated at the junction of middle and lower thirds without the loss of more than two or three drachms of blood. The shock was slight and the patient promptly rallied.

Dr. W. feels that he cannot too often impress the importance and value of the muriated tincture of iron as a means of arresting hemorrhage from the larger arteries. He contends that those who have failed to arrest hemorrhage in the large arteries with the tincture of iron have not used it properly; that they used it as in the first application in the case reported to-night, the iron being applied to the clot or disorganized tissues, and not to the artery itself. He says the tincture must be applied to the vessel itself, and to the surrounding tissues, else the important action of the styptic is lost, and we have nothing more than the formation of a clot, as by any other styptic.

In answer to a question as to his opinion of the character of the disease, he said that he was not decided in his opinion, but was disposed to regard it as malignant.

Dr. Battey had observed the prompt healing of an amputated thigh in a case of malignant disease of the inguinal glands, where the glands ulcerated, and the ulceration extended to the femoral artery, which, being opened, the patient speedily succumbed by hemorrhage. Adjourned.

